# ADAMTS-1: a novel target gene of an estrogen-induced transcription factor, EGR1, critical for embryo implantation in the mouse uterus

**DOI:** 10.1186/s13578-021-00672-8

**Published:** 2021-08-04

**Authors:** Mira Park, So Hee Park, Hyunsun Park, Hye-Ryun Kim, Hyunjung J. Lim, Haengseok Song

**Affiliations:** 1 Department of Biomedical Science, CHA University, Seongnam, Gyeonggi-do 13488 Republic of Korea; 2 Department of Veterinary Medicine, School of Veterinary Medicine, Konkuk University, 120 Neungdong-ro, Gwangjin-gu, Seoul, 05029 South Korea

**Keywords:** Estrogen, EGR1, ADAMTS-1, Transcription, Uterus

## Abstract

**Background:**

Recently, we demonstrated that estrogen (E_2_) induces early growth response 1 *(Egr1)* to mediate its actions on the uterine epithelium by controlling progesterone receptor signaling for successful embryo implantation. EGR1 is a transcription factor that regulates the spectrum of target genes in many different tissues, including the uterus. E_2_-induced EGR1 regulates a set of genes involved in epithelial cell remodeling during embryo implantation in the uterus. However, only few target genes of EGR1 in the uterus have been identified.

**Result:**

The expression of ADAM metallopeptidase with thrombospondin type 1 motif 1 (*Adamts-1*) was significantly downregulated in the uteri of E_2_-treated ovariectomized (OVX) *Egr1(−/−)* mice. Immunostaining of ADAMTS-1 revealed its exclusive expression in the uterine epithelium of OVX wild-type but not *Egr1(−/−)* mice treated with E_2_. The expression profiles of *Adamts-1* and *Egr1* were similar in the uteri of E_2_-treated OVX mice at various time points tested. Pre-treatment with ICI 182, 780, a nuclear estrogen receptor (ER) antagonist, effectively inhibited the E_2_-dependent induction of *Egr1* and *Adamts-1*. Pharmacologic inhibition of E_2_-induced ERK1/2 or p38 phosphorylation interfered with the induction of EGR1 and ADAMTS-1. Furthermore, ADAMTS-1, as well as EGR1, was induced in stroma cells surrounding the implanting blastocyst during embryo implantation. Transient transfection with EGR1 expression vectors significantly induced the expression of ADAMTS-1. Luciferase activity of the *Adamts-1* promoter containing EGR1 binding sites (EBSs) was increased by EGR1 in a dose-dependent manner, suggesting functional regulation of *Adamts-1* transcription by EGR1. Site-directed mutagenesis of EBS on the *Adamts-1* promoter demonstrated that EGR1 directly binds to the EBS at -1151/-1134 among four putative EBSs.

**Conclusions:**

Collectively, we have demonstrated that *Adamts-1* is a novel target gene of E_2_-ER-MAPK-EGR1, which is critical for embryo implantation in the mouse uterus during early pregnancy.

## Background

Early growth response 1 (*Egr1)* was initially known as an immediate-early response gene that is induced by various stress signals, including cytokines, growth factors, hormones, and DNA-damaging agents [1]. EGR1 as a transcription factor recognizes a highly conserved GC-rich promoter consensus motif on its target genes, such as *Tnf-α*, *Pten*, and *Socs-1* [2, 3]. It functions either as a tumor suppressor or oncogene, depending on the cell type and environmental conditions [4]. Several growth factors, such as *Igf-II*, *Pdgf-A*, and *Tgf-β1,* have been identified as direct targets of EGR1 in various tissues and pathological contexts including cancers [5–8]. Under normal conditions, EGR1 participates in the transcriptional regulation of several clock genes, such as *Per1*, *Per2*, and *Bmal1* [2], and is required for the transcriptional activation of *MMP1* during damaged tissue remodeling [9]. Collectively, the pleiotropic actions of EGR1 are brought about through many target genes that act as key factors in various physiological and pathological conditions. However, only few target genes of EGR1 in the uterus have been identified.

The balanced function of ovarian estrogen (E_2_) and progesterone (P_4_) is critical for successful pregnancy following embryo implantation [10]. Uncontrolled estrogenic activity could be a major cause of endometrial disorders, including infertility and cancers; however, only a handful of the downstream signaling factors of E_2_ have been identified in the uterus [11–13]. E_2_ induces the transcription factor EGR1 to fine-tune its functions that are responsible for uterine receptivity during embryo implantation [12, 14–16]. In addition, E_2_ promotes ERK1/2-dependent activation of ELK-1 to induce EGR1 the in MCF-7 breast cancer cells [17, 18]. While *Egr1(−/−)* mice showed normal ovulation, fertilization, and embryo development if exogenous gonadotrophins were administered, they completely failed in embryo implantation [12, 19]. To understand the function(s) of EGR1 as a key mediator of E_2_ in the uterus, the identification of the genes directly targeted by EGR1 is critical. By performing multi-step in silico promoter analyses using mRNA microarray data, we identified some of the potential target genes of EGR1, such as *Egr2*, *c-Kit*, and *Gadd45g*, in the mouse uterus [20]. We recently demonstrated that EGR1 transcriptionally regulates *c-Kit* expression to maintain uterine receptivity for embryo implantation in the mouse uterus [21]. A disintegrin and metalloproteinase with thrombospondin motifs 1 (*Adamts-1*), a new member of the ADAM-related proteins family, plays an key role in normal growth, organogenesis, and fertility [22, 23]. Although *Adamts-1* is a well-known target of P_4_-progesterone receptor (PR) signaling in the ovary during ovulation [24], the molecular mechanisms by which ovarian steroids regulate the transcription of *Adamts-1* in the uterus are poorly understood. Using multiple molecular and histological approaches, we have shown that EGR1, under the control of E_2_, transcriptionally regulates the expression of *Adamts-1* in the mouse uterus.

## Results

### Expression of *Adamts-1 *is dependent on E_2_-induced EGR1 in the uterine epithelium

**Fig. 1:**
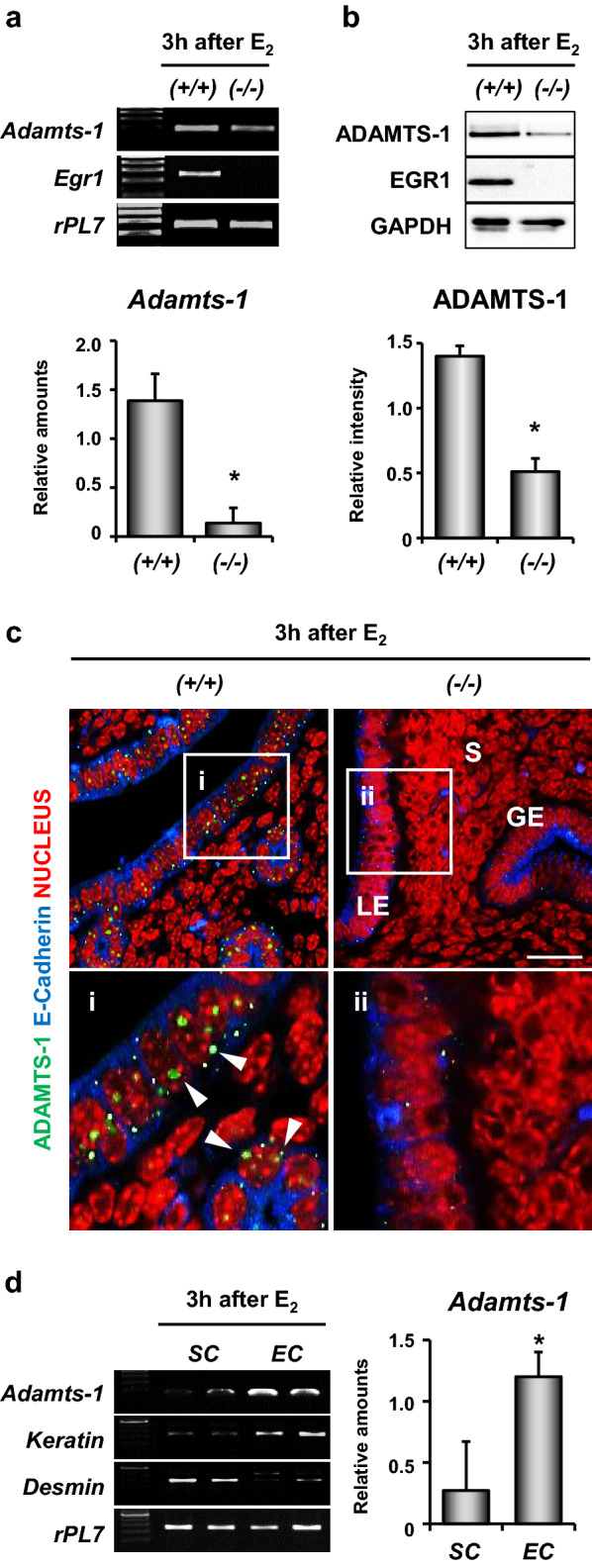
*Adamts-1* expression is significantly reduced in the uteri of *Egr1(−/−)* mice.** a** RT-PCR and real-time RT-PCR analyses of the relative amounts of *Adamts-1* and *Egr1* mRNAs in the uterus (4–5 mice in each group). **b** Western blot analyses of ADAMTS-1 and EGR1 expression (3–4 mice in each group). Error bars represent standard deviation. *P < 0.05. **c** Immunofluorescence staining of ADAMTS-1 showed that its expression was significantly reduced in the uteri of *Egr1(−/−)* mice. The second panels (i and ii) show the enlarged images of the boxed areas in the first panels. White arrowheads indicate ADAMTS-1 positive cells. Three independent fields were analyzed for each mouse (2–3 mice in each group). Green, blue, and red colors indicate ADAMTS-1, E-cadherin, and nucleus, respectively. *GE* glandular epithelial cells, *LE* luminal epithelial cells, *S* stromal cells. Scale Bar: 25 µm. **d** RT-PCR and real-time RT-PCR analyses of *Adamts-1* mRNAs in the isolated uterine stromal cells (SC) and epithelial cells (EC). *Keratin* and *Desmin* are EC and SC marker genes, respectively (six mice in each group). *P < 0.05

Previously, in silico promoter analyses of the microarray data from *Egr1(*+*/*+*)* and *Egr1(−/−)* mice uteri revealed putative EGR1 binding sites (EBSs) in the *Adamts-1* promoter [20]. Thus, we examined if *Adamts-1* was a direct target gene of the transcription factor, EGR1, in the uterus. The results of reverse transcription (RT)-PCR and real-time RT-PCR showed that the expression of *Adamts-1* was significantly reduced in the uteri of *Egr1(−/−)* mice treated with E_2_ for 3 h (Fig. 1a). Western blot analyses also showed that the expression levels of both EGR1 and ADAMTS-1 were significantly reduced in *Egr1(−/−)* mice (Fig. 1b). Immunofluorescence staining of ADAMTS-1 showed its localization in the uterine epithelial cells of wild-type mice, but not *Egr1(−/−)* mice, at 3 h post E_2_ treatment (Fig. 1c). To investigate the expression of *Adamts-1* in uterine cell type, we isolate the epithelial and stromal cells. The results of RT-PCR and real-time RT-PCR showed that the expression of *Adamts-1* was significantly increased in the epithelial cells of the uteri treated with E_2_ for 3 h (Fig. 1d).

### *Adamts-1* and *Egr1* are rapidly and transiently induced by E_2_ in the mouse uterus

**Fig. 2:**
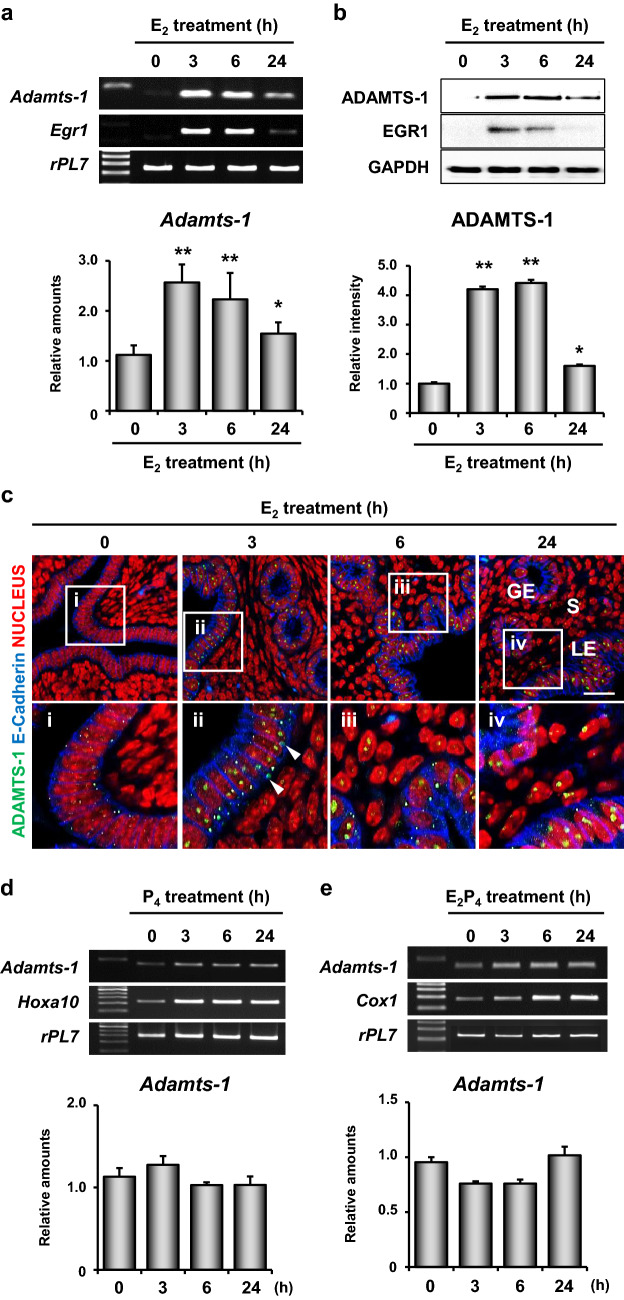
Time-dependent expression of *Adamts-1* induced by E_2_ in the mouse uterus. **a**, **b** Spatiotemporal expression patterns of *Adamts-1* and *Egr1* in the uterus at several time points after E_2_ treatment. RT-PCR and real-time RT-PCR **a** and Western blot analyses **b** (4–5 mice in each group). Error bars represent standard deviation. *P < 0.05, **P < 0.01. **c** Immunofluorescence staining of ADAMTS-1 showed that ADAMTS-1 is specifically localized in the uterine epithelium (white arrowheads). The second panels (i to iv) show the enlarged images of the boxed areas in the first panels. Three independent fields were analyzed for each mouse (2–3 mice in each group). Green, blue, and red colors indicate ADAMTS-1, E-cadherin, and nucleus, respectively. GE, glandular epithelial cells; LE, luminal epithelial cells; S, stromal cells. Scale Bar: 2 µm. RT-PCR and real-time RT-PCR were performed to analyze the relative levels of *Adamts-1* induced in the uteri of OVX mice by P_4_ alone **d** or by a combination of E_2_ + P_4_ **e** at different time points post hormone treatment (4–5 mice for each time point). *Hoxa10* and *Cox1* represent temporal expression markers for the time-dependent hormonal response. *rPL7* represents the loading control

To further examine the mechanisms underlying the regulation of *Adamts-1* expression by E_2_-induced EGR1 in the uterus, we investigated the spatiotemporal expression patterns of *Adamts-1* in the uteri of ovariectomized (OVX) mice treated with E_2_ for 0, 3, 6, or 24 h following hormone treatment. RT-PCR and real-time RT-PCR analyses demonstrated that the expression pattern of *Adamts-1* was similar to that of *Egr1* in the uterus (Fig. 2a). Both *Egr1* and *Adamts-1* were transiently and rapidly induced by E_2_, with a peak at 3 h post E_2_ treatment. Western blot analyses revealed the unique expression pattern of ADAMTS-1 induced by E_2_ (Fig. 2b). Immunofluorescence staining showed that ADAMTS-1 was predominantly localized in the epithelial cells of the uterus (arrowheads in Fig. 2c).

To investigate the effects of P_4_ on *Adamts-1* expression in the mouse uterus, P_4_ alone or a combination of E_2_ + P_4_ was administered to mice OVX at various time points following hormone treatment. We found that P_4_ alone did not induce the expression of *Adamts-1* in the uteri of OVX mice (Fig. 2d). However, when a combination of E_2_ + P_4_ was used, P_4_ effectively inhibited the E_2_-induced expression of *Adamts-1* in the uterus (Fig. 2e).

### Activation of ERK1/2 and p38 MAPK by the E_2_-ER(s) pathways is required for the EGR1-dependent induction of ADAMTS-1 in the uterus

**Fig. 3:**
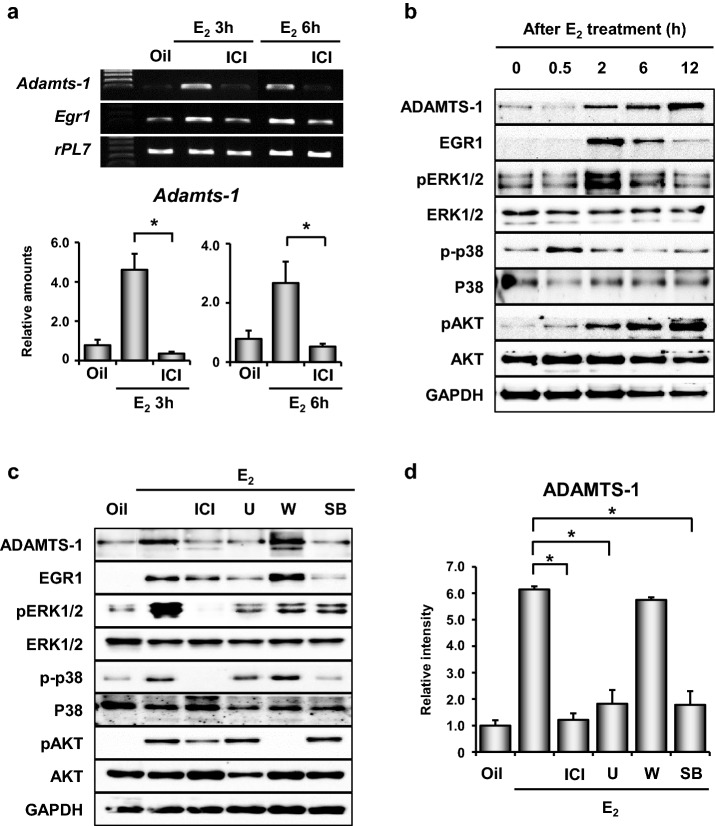
E_2_ regulates *Adamts-1* expression via nuclear ER(s) in the uterus.** a** RT-PCR and real-time RT-PCR analyses of the expressions of *Adamts-1* and *Egr1* mRNAs in the uteri of adult OVX mice pre-treated with an ER antagonist, ICI 182,780 (500 µg/mouse) 30 min before E_2_ injection (200 ng/mouse) (4–5 mice in group). **b** Representative images of Western blot analyses examining the rapid changes in the phosphorylation status of AKT and MAPKs (ERK1/2 and p38) by E_2_ (3–4 mice in group). **C** Pharmacological inhibition of E_2_-dependent ADAMTS-1 and EGR1 induction by inhibitors of AKT (W; wortmannin, 14 μg/mouse), ERK 1/2 (U; U0126, 160 mg/kg), or p38 (SB; SB203580, 1 mg/mouse). Each inhibitor was administered to OVX mice 30 min before E_2_ administration (3–4 mice in each group). **d** Relative intensity of ADAMTS-1 protein expression *P < 0.05

To determine whether E_2_ induces *Egr1* and *Adamts-1* expression via the activation of its nuclear estrogen receptor (ER)α and ERβ, in the mouse uterus, we examined the expression levels of *Adamts-1* in OVX mice pre-treated with ICI 182,780 (an ER antagonist), 30 min before E_2_ injection. At 2 and 4 h post-E_2_ injection, the E_2_-dependent expression of *Adamts-1* and *Egr1* mRNAs was profoundly reduced in the uteri of OVX mice pre-treated with ICI 182,780 (Fig. 3a). E_2_ exerts its functions through both genomic and non-genomic pathways, such as MAPK pathways, which regulate EGR1 expression in various cell types [16, 25, 26]. Thus, to determine if the non-genomic function(s) of E_2_ is/are required for the induction of ADAMTS-1 in the uterus, we evaluated the phosphorylation patterns of the MAPK and AKT pathways in the uteri of OVX mice that were treated with E_2_ for various time periods. While E_2_ gradually increased the phosphorylation of AKT and JNK, ERK1/2 and p38 were rapidly activated, with a peak at 2 h post E_2_ treatment (Fig. 3b). ICI 182,780 significantly inhibited the E_2_-induced phosphorylation of AKT, ERK1/2, and p38. Pre-treatment with the pharmacological inhibitors of each kinase showed that the E_2_-ER-dependent phosphorylation of p38 and ERK1/2 was required for the induction of EGR1 and ADAMTS-1 in the uterus (Fig. 3c, d). These results suggest that the transcription of *Adamts-1* could be regulated by the E_2_-ER-ERK/p38-EGR1 pathway in the mouse uterus.

### ADAMTS-1 and EGR1 are induced in the stromal cells surrounding the implanting blastocyst during embryo implantation

**Fig. 4:**
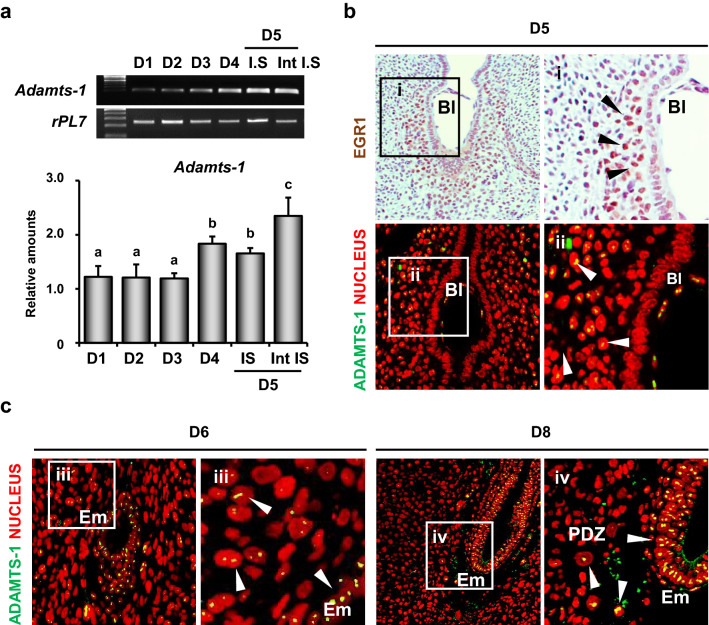
The expression of *Adamts-1* in the stromal cells surrounding the implanting blastocyst during embryo implantation.** a** RT-PCR and real-time RT-PCR of *Adamts-1* expression in the uterus during pregnancy (4–6 mice for each day of pregnancy). *rPL7* represents the loading control. *P < 0.05. **b**, **c** Immunostaining of ADAMTS-1 in uterine tissues. The second panels (i to iv) show the enlarged images of the boxed areas in the first panels. White arrowheads indicate ADAMTS-1 positive cells. Three independent fields were analyzed for each mouse (3–4 mice for each day). Green and red colors indicate ADAMTS-1 and nucleus, respectively. *GE* glandular epithelial cells, *LE* luminal epithelial cells, *S* stromal cells, *Bl* Blastocyst, *Em* embryo, *PDZ* primary decidual zone. Scale Bar: 2 µm

During early pregnancy, we found that the expression level of *Adamts-1* mRNA increased on the day 4 of pregnancy (D4) (Fig. 4a). Previously, we had demonstrated that EGR1 was exclusively induced in the stromal cells surrounding the implanting blastocyst (Bl) during mice embryo implantation [12]. Immunostaining of EGR1 and ADAMTS-1 in the uterus containing the implantation sites on D5 showed that both EGR1 and ADAMTS-1 were induced in the decidualizing stromal cells during embryo implantation (right panels in Fig. 4b). Furthermore, ADAMTS-1 was also found in the trophoblasts of the Bl (yellow dots in BI). However, ADAMTS-1, but not EGR1, was found in a subset of decidualizing cells in the primary decidual zone (PDZ) on D6 and D8 (Fig. 4c).

### The effective EBS is present within the distal region of the *Adamts-1* promoter

**Fig. 5:**
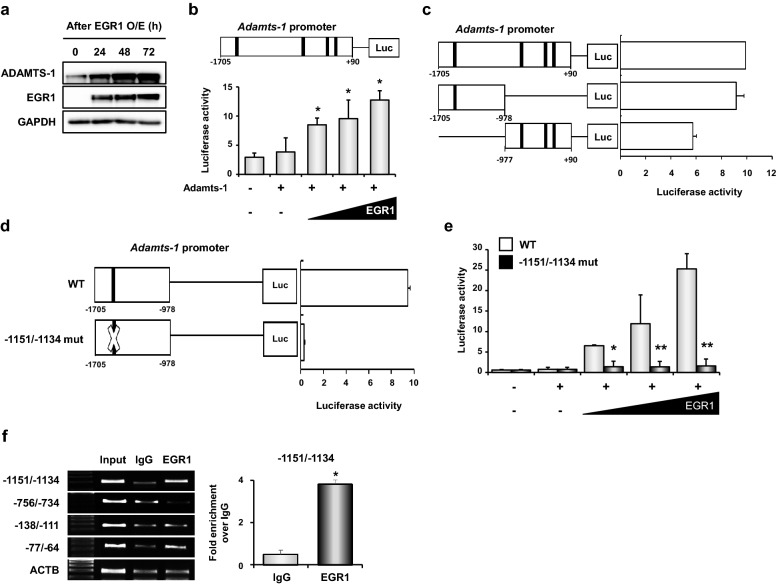
The functional EGR1 binding site is located in the distal region of the *Adamts-1* promoter.** a** Western blot analyses of the expression profiles of ADAMTS-1 and EGR1 after transfection of the EGR1 expression vector. GAPDH represents the loading control. **b** *Adamts-1* luciferase vector (− 1705/+ 90) was co-transfected with increasing concentrations of the EGR1 expression vector (pIRES dsRED2/EGR1) in 293 T cells, as indicated. At 48 h post transfection, the cells were collected, and the luciferase activity was measured. **c** EGR1 expression vector was transiently co-transfected with one of three different *Adamts-1* promoter constructs in 293 T cells. The luciferase activity was measured at 48 h post transfection. The firefly activity was normalized to the activity of Renilla luciferase, and the luciferase activity of the untreated cells was designated as one relative value. **d** EGR1 expression vector was co-transfected in 293 T cells along with one of two different *Adamts-1* promoter constructs containing mutations in EGR1 binding sites. The luciferase activity was measured at 48 h post transfection. **e** Luciferase activity of the WT and mutant (− 1151/− 1134) *Adamts-1* promoters co-transfected with an increasing amount of EGR1 expression vector in 293 T cells is indicated. Luciferase activity was calculated relative to the expression of the pGL4.10 basic vector, which served as the negative control. *P < 0.05, **P < 0.01*.* **f** Chromatin immunoprecipitation (ChIP)-PCR and real-time ChIP-PCR for four putative EBS in the *Adamts-1* promoter. The EGR1-MYC-FLAG expression vector was transfected into 293 T cells and genomic DNA was used for ChIP-PCR. A schematic cartoon (right panel in **d**) to show locations of four different primers on the *Adamts-1* promoter to amplify genomic DNAs precipitated by FLAG antibody. Note that the genomic DNA containing − 1151/− 1134 of the *Adamts-1* promoter was significantly enriched. *P < *0.05*

To further understand the molecular interaction(s) of EGR1 with the *Adamts-1* promoter, we performed a series of luciferase promoter-reporter assays. First, we examined whether the forced expression of EGR1 induced ADAMTS-1 expression in 293 T cells. As shown in Fig. 5a, Western blot analyses clearly showed that transfection with an EGR1 overexpression vector significantly increased the expression of ADAMTS-1 and EGR1 in a time-dependent manner. We then performed luciferase assays on a construct with a region of the *Adamts-1* promoter region (− 1705 to + 90) containing four putative EBS. We found that the luciferase activity of the *Adamts-1* promoter was increased by EGR1 expression vectors in a dose-dependent manner (Fig. 5b), suggesting the presence of EBS in the *Adamts-1* promoter. Furthermore, we found that the distal 1 kb region of the *Adamts-1* promoter (− 1705 to − 978 from the transcription start site) contained one EBS (− 1151/− 1134) that was sufficient for the EGR1-dependent activation of the *Adamts-1* promoter (Fig. 5c). To further examine the function of the EBS at the distal region of the *Adamts-1* promoter, luciferase assays were performed on a mutant EBS containing the *Adamts-1* promoter. A mutation (mut) at the − 1151/− 1134 of the EBS completely destroyed the transcriptional activity of the plasmid construct (Fig. 5d). We also found that EGR1 did not activate the distal 1 kb region of the *Adamts-1* promoter containing a − 1151/− 1134 mut, although the wild-type distal promoter region responded to EGR1 in a dose-dependent manner (Fig. 5e). To elucidate the physical interaction between EGR1 and − 1151/− 1134 EBS of the *Adamts-1* promoter, a FLAG-tagged EGR1 expression vector was transfected into 293 T cells and chromatin immunoprecipitation (ChIP) for FLAG was performed. ChIP-PCR and ChIP-real-time PCR for each EBS provided evidence that − 1151/− 1134 EBS was critical for EGR1-dependent *Adamts-1* transcription (Fig. 5f).

## Discussion

Previously, we had demonstrated that E_2_ induces the transcription factor EGR1, to fine-tune its major effects on the uterine epithelium during embryo implantation [12]. EGR1 governs the expression of an array of genes regulated by E_2_ in the uterus. Our in silico analyses suggested that EGR1 physically binds to the *Adamts-1* promoter and induces its expression in the uterus [20]. Using multiple approaches, we have shown that EGR1 regulates the expression of *Adamts-1* at the transcriptional level in the uterus. EGR1 is rapidly and transiently induced by the E_2_-induced phosphorylation of p38 and ERK1/2 in the uterus [21]. It is also induced in the ovary immediately after ovulation by a surge of luteinizing hormone (LH) [27]. A recent study suggested that LH-dependent ERK1/2 signaling promotes the simultaneous induction of *Egr1* and *Adamts-1* in bovine granulosa cells [28]. Interestingly, while the expression of *Adamts-1* was significantly reduced in the uterus of *Egr1*(−/−) mice (Fig. 1), its expression was not altered in the ovary of *Egr1*(−/−) mice following the LH surge (unpublished observation). In addition, we found that the expression of *Adamts-1* coincided with that of *Egr1* in the uterus of mice treated with E_2_ (Fig. 2). However, further studies are warranted to understand the different regulatory mechanisms by which EGR1 interacts with the *Adamts-1* promoter in different cellular contexts in the uterus and ovary of mice.

ADAMTS-1 is a protease that mediates follicular rupture [29]. At the time of ovulation, it cleaves the extracellular matrix of the thecal cell layer, thereby allowing the release of the cumulus oocyte complexes from the ovary [30]. In addition, it facilitates the hormonal response of granulosa cells by cleaving the proteoglycans that inhibit the binding of gonadotrophins to their receptors [23, 29]. Although *Adamts-1* is a well-known target of the P_4_-PR signaling pathway in the ovary, during ovulation, its expression in the uterus is strictly regulated by E_2_, and not by P_4_. We found that the P_4_-PR signaling pathway alone did not affect the expression of *Adamts-1* in the uterus (Fig. 2d). Rather, it interfered with the effects of E_2_ on *Egr1* expression, leading to a reduction in *Adamts-1* expression in the uterus (Fig. 2e). Consistent with a previous study [31], we found that the expression of *Admats-1* increased during the proestrus and estrus stages when E_2_ is the dominant hormone (data not shown), suggesting that *Adamts-1* is mainly regulated by E_2_ in the uterus. However, a previous report showed that the expression of *Adamts-1* mRNA was not significantly altered in the bovine endometrium during the estrous cycle [32]. In addition, other studies have revealed an increase in the expression of *Adamts-1* mRNA by P_4_ in cultured bovine and human stromal cells [32, 33]. Furthermore, E_2_ attenuated the positive effects of P_4_ on *Adamts-1* in a concentration-dependent manner [33]. This discrepancy could be derived between from species differences and/or different cellular/physiological contexts.

Endometrial stromal cells undergo extensive remodeling during decidualization [34, 35]. Decidualization response is partially impaired in *Egr1*(−/−) mice [12], and EGR1 transcript levels are downregulated in the endometrium of patients with repeated implantation failure [36]. Furthermore, EGR1 is required to transcriptionally program pre-decidual human endometrial stromal cells for decidualization and its expression levels are required to be reduced to enable decidualization [36, 37]. This down-regulation of EGR1 in human decidualized stromal cells is consistent with our results that the expression of EGR1 is reduced in the PDZ of mice on D6 and D8 (Fig. 4c). However, ADAMTS-1 was persistently maintained in these cells on D6 and D8, suggesting the presence of other regulatory mechanisms that affect ADAMTS-1 expression during this event. ADAMTS-1, which is responsible for extracellular matrix remodeling, is known to play a crucial role in initiating and successfully maintaining decidualization [31–33]. However, *Adamts-1*(−/−) mice appear normal with respect to embryo implantation and decidualization [22, 23, 38]. This could be due to the redundant expression of other metalloproteases in the uterus during embryo implantation and decidualization [39–41]. In fact, not only ADAMTS-1 but also ADAMTS-5 proteins were identified in human decidualized stromal cells [40].

Using in silico analyses, we found four putative EBS within − 2 kb of the *Adamts-1* promoter (Fig. 5b). Previous studies, including ours, have shown that EBS are enriched within − 500 of the promoters of genes whose expressions are influenced by EGR1 [20, 42, 43]. For example, in many species, EBS is found within − 500 of the *LH-β* subunit promoter [19]. Moreover, EGR1 directly interacts with an EBS in the promoter regions of *MMP9* (− 569/− 553) and *MMP1* (− 137/− 119) in HeLa cells [9, 44]. Although a putative EBS at − 133/− 122 of the *Adamts-1* promoter is conserved among many species, including humans, mice, and rats [20], we found that EGR1 does not interact with this EBS. Instead, we found that EGR1 binds to the EBS at the − 1151/− 1134 region of the *Adamts-1* promoter, the mutation of which exclusively abrogated the transcriptional activity of EGR1 (Fig. 5). While we cannot exclude that the other EBS present within − 1 kb of the *Adamts-1* promoter can play a role in the EGR1-dependent transcription of *Adamts-1*, our results suggest that EGR1 physically interacts with the *Adamts-1* promoter at the − 1151/− 1134 region and enhances its expression.

## Conclusion

We have demonstrated that the transcription of *Adamts-1* is regulated exclusively by the E_2_-dependent EGR1 transcription factor in the uterus, whereas by the P_4_ signaling pathway in the ovary in mice. Collectively, *Adamts-1,* whose expression is localized in the uterine epithelium, is a novel target gene of E_2_-ER-MAPK-EGR1, critical for embryo implantation in the mouse uterus during early pregnancy.

## Methods

### Reagents and chemicals

E_2_ (17β-estradiol; Sigma-Aldrich, St. Louis, MO, USA), P_4_ (Sigma-Aldrich), ICI 182,780 (Sigma-Aldrich), U0126 (MEK 1/2 inhibitor; Cell Signaling Technology, Danvers, MA, USA), wortmannin (AKT inhibitor; Cell signaling Technology), and SB203580 (p38 inhibitor; Selleck Chemicals, Houston, TX, USA) were used in this study.

### Animals

All mice were housed following the institutional guidelines for laboratory animals (Animal Care Facility of CHA University). This study was approved by the Institutional Animal Care and Use Committee (IACUC, Approval Number: 190168). Adult ICR mice (8-week-old), provided by KOATECH (Pyeontaek, Gyeonggi, Korea) were housed under temperature- and light-controlled conditions for 12 h daily and fed ad libitum. *Egr1*(−/−) mice were kindly provided by Dr. Jeffrey Milbrandt (Washington University, St. Louis, MO, USA). For genotyping, PCR analysis of tail genomic DNA was used, as previously described [12, 19].

### Hormone treatments

To examine the effects of ovarian steroid hormones on *Adamts-1* expression, adult female mice were OVX, allowed to rest for 10 days, and subsequently provided the necessary treatments for each experiment performed in the study. The mice were sacrificed, and their uterine tissues were collected for molecular and histological analyses. RNA or protein extractions were carried out following the injection of E_2_ and/or P_4_. To investigate the time-dependent action of E_2_ on the expression of *Adamts-1* in the mouse uterus, OVX mice were subcutaneously injected with either the vehicle (sesame oil, 0.1 mL) or E_2_ (200 ng/mouse), and sacrificed at various time points (0.5–12 h) following the injection. To analyze whether E_2_ functions through the nuclear ER to induce *Adamts-1* expression in the mouse uterus, adult OVX mice were pre-treated with ICI 182, 780 (500 μg/mouse), an ER antagonist, and subsequently treated with P_4_ (2 mg/mouse) alone or P_4_ + E_2_. To determine the signaling pathway(s) activated by the non-genomic effect(s) of E_2_ on *Adamts-1* induction in the mouse uterus, adult OVX mice were pre-treated with the pharmacological inhibitors, U0126 (160 mg/kg), wortmannin (14 µg/mouse), and SB203580 (1 mg/mouse), in combination with E_2_.

### RNA preparation, RT-PCR, and real-time RT-PCR

Mouse uteri (from 3 to 5 mice in experimental group) were collected and immediately frozen in liquid nitrogen. Subsequently, they were individually prepared for protein and/or total RNA extraction. Total RNA was extracted individually from each uterine tissue using TRIzol Reagent (Ambion, Carlsbad, CA, USA). Two micrograms of uterine total RNA were subjected to RT using M-MLV reverse transcriptase (Promega, Madison, WI, USA), random primers, and oligo dT, to obtain cDNA. The cDNA obtained was used as the template in a PCR by using specific primers and optimized time–temperature cycles. Real-time RT-PCR was performed by using the iQ™ SYBR^®^ Green Supermix (Bio-Rad), and by monitoring the increase in the fluorescence of the SYBR Green dye in real-time, as previously described on a real-time PCR detection system (Bio-Rad) [16, 45]. To compare transcript levels between samples, a standard curve was prepared using several serial dilutions of the cDNA sample. To calculate the relative abundance of each gene, this standard curve was used. The C_t_ values were normalized to the relative amounts of the ribosomal protein, L7 (*rPL7*) [12]. All PCR reactions were performed in duplicate.

### Western blot analysis

Uterine samples were homogenized using a Polytron homogenizer (Brinkmann, Westbury, NY, USA). The protein was extracted using PRO-PREP Protein Extraction Solution (iNtRON Biotechnology, Seongnam, Gyeonggi, Korea) containing 1X phosphatase inhibitor (Roche Applied Sciences, Indianapolis, IN, USA) by lysing the cells. The protein extracts were separated by SDS-PAGE (8–10%) and transferred to a nitrocellulose membrane (Bio-Rad). Subsequently, the membranes were subjected to Western blot analyses with anti-ADAMTS-1 (Abcam, Cambridge, UK, ab39194, 1:1000), anti-EGR1 (Cell Signaling, #4153, 1:1000), anti-pAKT (Cell Signaling, #9271, 1:1000), anti-AKT (Cell signaling, #9272, 1:1000), anti-pERK1/2 (Cell Signaling, #9101, 1:1000), anti-ERK1/2 (Cell Signaling, #9102, 1:1000), anti-p-p38 (Santa Cruz, sc-17852, 1:1000), anti-p38 (Ab frontier, LF-MA0126, 1:1000), anti-pJNK1/2 (Santa Cruz, 1:1000), and anti-GAPDH (Cell Signaling, #21,118, 1:2000) antibodies. The secondary antibodies were HRP-conjugated goat anti-rabbit or mouse (Invitrogen, Carlsbad, CA, USA, 1:3000). Immunoreactive bands were detected using the Immune-Star Western™ Chemiluminescence Kit (Bio-Rad). The chemiluminescence signal was detected using the ChemiDOC™ XRS + System (Bio-Rad).

### Immunofluorescence

To determine the cell-type specific localization of ADAMTS-1 following E_2_ treatment, the uteri were fixed in 4% paraformaldehyde and embedded in paraplast (Leica Biosystems, St. Louis LLC, Diemen, Netherlands). Uterine Sects. (5 µm) were de-paraffinized and rehydrated, and the endogenous peroxidase was inactivated using 3% H_2_O_2_. The tissue sections were subjected to antigen retrieval by immersing in sodium citrate buffer (10 mM, pH 6.0) for 20 min. Serum as a protein block (Dako, Carpinteria, CA, USA) was used to block non-specific staining. The tissue sections were then incubated with primary anti-ADAMTS-1 antibody (Abcam, 1:100) at 4 °C overnight. The secondary antibodies were FITC-conjugated anti-goat (Jackson ImmunoResearch, West Grove, PA, 1:250) for 60 min at room temperature. The sections were counter-stained with TO-PRO-3-iodide (Life Technologies, Carlsbad, CA, USA, 1:400). Microscopic images were obtained and analyzed using ZEN software (ZEISS, Oberkochen, Germany).

### Isolation of uterine epithelial and stromal cells

Uteri were dissected from 6 female mice at 3 h after E_2_ treatment, minced into small pieces, and incubated in HBSS (Thermo Fisher Scientific) containing dispase (2.4 U/ml) and pancreatin (25 mg/ml) for 1 h at 4 °C and 1 h at room temperature. Tissues were incubated at 37 °C for 10 min, then the supernatant the epithelial cell-rich fraction was collected. The remaining stromal cell-rich pellet was then digested with collagenase (0.5 mg/ml) after filtration through a 70-µm nylon mesh. The epithelial and stromal cells were prepared for total RNA extraction.

### In silico promoter* analysis*

The promoter sequence of *Adamts-1,* from − 2000 to + 200, was obtained from the UCSC gene sorter (http://genome.ucsc.edu), and the putative EBSs in the *Adamts-1* promoter were identified using Promo (http://alggen.lsi.upc.es).

### Promoter reporter assay

The *Adamts-1* promoter region, ranging from − 1705 to + 90, was amplified from mouse genomic DNA by PCR with Forward (5′-ACT GTG GAT GTC AGT GAG AGC-3′) and Reverse (5′-GCT GCT TTC TAG CGA GTG CAA-3′) primers. The amplified PCR product was cloned into the pGL4.10-basic reporter, and the resulting plasmid was designated as pGL4. 10/*Adamts-1* (− 1705/+ 90). Reporter vectors containing the *Adamts-1* promoter region from − 1705/− 978 or − 977/+ 90 were generated using pGL4.10/*Adamts-1*. Mutant promoter constructs (− 1151/− 1134 mut) were produced using the EZ change™ site-directed mutagenesis kit (Enzynomics, Seoul, Korea). In 12-well plates, 293 T cells were seeded and transfected with 2 μg of the *Adamts-1* promoter-reporter construct and EGR1 expression vector (pIRES dsRED2/EGR1) using Gene Porter 3000 (Genlantis, San Diego, CA, USA) transfection reagents, following the manufacturer’s instructions. A pRL-null plasmid (50 ng) encoding Renilla luciferase was incorporated in all samples to monitor the transfection efficiency. After 48 h, the firefly and Renilla luciferase activities were measured sequentially from a single sample using the Dual-Glo™ Luciferase Assay System (Promega, Madison, WI, USA) using an illuminometer.

### Chromatin immunoprecipitation (ChIP)

Human EGR1 was amplified and cloned into the pCMV6-ACIRES-GFP-Puro vector to tag EGR1 with Myc and FLAG (Origene Technologies, Rockville, MD, USA). 293 T cells were transfected with an EGR1-MYC-FLAG expression plasmid for 48 h, washed once with PBS, and added to 10 ml of PBS containing 1% formaldehyde to covalently cross-link any DNA–protein complexes at room temperature for 10 min. The beads were added to the samples, rotated for 30 min, and collected by centrifugation at 12,000×*g* for 1 min. The elution buffers were added to the samples and the supernatants were transferred to clean microcentrifuge tubes. The DNA samples were used for PCR reactions with the appropriate primers.

### Statistics

Each experiment was performed at least three times. Data were presented as mean ± S.D. GraphPad Prism ver. 8 software (GraphPad Software, La Jolla, CA, USA) was used to perform statistical analyses for the Mann–Whitney *U* test. Statistical significance was set at *P* < 0.05.

## Data Availability

All data generated or analyzed during this study are included in this published article.

## Abbreviations

E_2_: Estrogen
P_4_: Progesterone
ER: Estrogen receptor
PR: Progesterone receptor
OVX: Ovariectomized
*Egr1*: Early growth response 1
*Adamts-1*: ADAM metallopeptidase with thrombospondin type 1 motif 1
SC: Stromal cell
EC: Epithelial cell
EBS: EGR1 binding site
RT: Reverse transcription
LE: Luminal epithelial cells
GE: Glandular epithelial cells
S: Stromal cells
Em: Embryo
Bl: Blastocyst
PDZ: Primary decidual zone
D4: Day 4 of pregnancy
ChIP: Chromatin immunoprecipitation
